# On Dysentery

**Published:** 1852-09

**Authors:** George S. Upshur

**Affiliations:** Fellow of the Medical Society of Virginia; Member of the American Medical Association, &c., Norfolk, Virginia


*On Dysentery.* By GEORGE S. UPSHUR, A. M., M. D., Fellow of Medical Society of Virginia; Member of the American Medial Association, &c. Norfolk, Virginia.

So much has been written upon dysentery, not only in the systematic works on Practice, but also in the various Medical Journals, that doubtless little is left to be said that has not already been said upon this subject. I do not propose, therefore, in this article, to write a treatise on this subject, nor to enter with any degree of minuteness into its causes, symptoms, or pathology, but simply to examine, as briefly as possible, the value of the many remedies that have from time to time been zealously urged upon our attention and to illustrate the whole, as far as my own experience goes, by the history of a few cases taken from every day practice.

Dysentery may be called a genuine *cosmopolite*, for it is confined to no fixed locality. Indeed, so common is the affection, that no physician, who has any business at all, fails to see it every week in the year. While it very frequently prevails as an epidemic, particularly amongst those who are subject to sudden vicissitudes of weather, and are accustomed to unwholesome food and ill-ventilated apartments, it cannot be said to be confined exclusively to any particular class or condition, but frequently attacks the high and the low together, and is found often as difficult of management in one as the other.

The most prolific *cause* of dysentery is undoubtedly a want of balance between the functions of the skin and the mucous surface of the bowels. No question in pathology is better established than that the skin is closely allied in its healthy and diseased action, to the intestinal mucous membrane, and indeed to all the mucous membranes, and that they frequently perform a vicarious part towards each other. Hence a cessation of the normal secretion from the skin is almost invariably followed by a congested state of the mucous surfaces, amongst which the mucous membrane of the intestines bears a conspicuous part. Sudden exposure then to drafts of cold air, when the patient is over-heated and sweating freely, is very apt to give an attack of the dysentery—especially if he should be much fatigued at the time. Irritating ingesta, unripe and acid fruits, and drastic cathartics are also frequent causes of the disease. I have also seen the disease produced in a number of instances by the too free use of capsicum, either as a condiment, or exhibited, combined with quinia, in intermittent fever.

Dysentery is one of the few frank, open inflammations of an inportant organ that does not make its attack by rigors. *Sometimes* it is ushered in by decided chill, but generally it comes on gradually;—at first there is simple looseness of the bowels, the dejections being large, semi-fluid, and accompanied with only a little griping pain along the track of the colon. These symptoms, however, are soon followed by fever, thirst, and nausea, with small and very frequent discharges of pure blood, or bloody mucus, and pain and straining at stool—these last symptoms often continue for several days after the fever has subsided and the appetite has returned. The disease is then apt to run into the chronic form, from which result ulceration of the bowel and a hypertrophied condition of the mucous membrane, which is very troublesome to the patient, and difficult of management.

The *pathology* of the disease is embraced in the simple remark that it is an inflammation of the *mucous* coat of the large intestine. When the muscular tunic and peritoneum are involved, it is to be considered merely a complication, *and no part of the disease proper.*

*Treatment.*

*Blood-letting.*—There are few cases of dysentery,—I do not speak now of the epidemic disease,—that do not require the *free use* of the lancet, if the patient is seen within the first five days after the attack. The state of the pulse is here of less value than in any of the phlegmasiæ, and indeed scarcely to be consulted in reference to the propriety of taking blood, *if the tormina, tenesmus, and frequent bloody or mucous stools* are prominent symptoms. It is well known that the pulse is rarely found to be full, hard and bounding, in inflammation, however acute, of the mucous membranes—on the contrary, it is apt to be small, frequent, and very compressible, especially in inflammation of the mucous coat of the bowels, and therefore is well calculated to mislead, if relied on solely, as is too often the case, to decide the question of blood-letting. The immediate and intimate connection of the bowels with the most important nervous centres—their abundant supply of blood-vessels, and the indispensable part they perform in the economy, would lead us to conclude, *a priori*, that, when inflamed, the force of the heart's action, and indeed all the vital powers would be depressed in a marked degree. And we find this to be the case in every day practice, but such depression should never deter us from the free use of the lancet, if other symptoms call for it. I would lay down the rule, then, that in all cases of dysentery in which there are frequent, small, bloody, or muco-bloody stools, accompanied by distressing tormina and tenesmus, if seen early, general bleeding *as far as the patient will bear it*, is absolutely demanded, no matter if the pulse does *seem* to contra-indicate it. We have only to reflect for a moment that we have here acute inflammation of an important organ, which it is necessary to arrest as soon as possible, and that the lancet is the great agent in the relief of all acute, frank inflammations, to be convinced of the propriety of the rule, without reference to the temporary depression of the pulse, produced by the intensity of the disease. I dwell upon this subject because I deem it of vital importance in the treatment of dysentery. I can call to mind at least half a dozen cases of death from this disease, in the commencement of my practice, which, after reflection, convinces me might possibly have been saved by copious blood-letting. The condition of the pulse alone guided me, without reference to other and more important symptoms. Doubtless many of my medical brethren have frequently committed the same blunder. In the treatment of disease, no single symptom, however important in itself, ought to be permitted to control the judgment of the physician. Yet how often does the pulse sit as judge and alone decide questions in therapeutics from which it would be considered by some the height of rashness to appeal.

After general blood-letting, the application of leeches or cups along the track of the colon, or of leeches to the anus, will be found of great service—but topical depletion should never be permitted in lieu of general depletion, where this last can be resorted to. Indeed, cups and leeches are of little value in the first stage of acute dysentery, unless preceded by the lancet.

*Mercurials.*—No agents have been more over-rated in the treatment of dysentery than mercurials. The combination of calomel, opium and ipecac. is the *alpha* and *omega* of therapeutics in this disease, with a very large and respectable body of physicians; and such is the testimony adduced in its favor that I doubt not beneficial results have *followed* its use in particular cases. I am disposed, however, to attribute the benefits to the *opium and ipecac. only*, and am satisfied from careful and ample observation, that they would have accomplished much more good without the calomel. It may be true that in the dysentery of tropical climates, where torpor of the liver is usually a complication, mercury is sometimes administered with benefit, but in the ordinary dysentery of this country I am persuaded it does more harm than good. My own observation in this regard is corroborated by some of the best authorities in medicine.

Dr. Cheyne, in 3d vol. Dublin Hospital Reports, says: "Mercury could not be depended upon, and did not relieve in numerous instances when the mouth was affected, and sometimes seemed to increase the disease."

M. Twining, who saw a vast amount of the disease in the General Hospital at Calcutta, says: "I speak without hesitation on this subject, from having too often seen the fallacy of trusting to the effects of calomel for the cure of severe acute dysentery, and having tried that medicine extensively in every form of the disease."*

Dr. Mackintosh, (*Practice of Physic*, vol. I. p. 366, 1837,) says: "My own experience in this country, as well as within the tropics, enables me to confirm the statement of Dr. Cheyne and M. Twining."

Dr. C. A. Lee, in a note to the art. *Dysentery*, in Copland's Dictionary, says: "With respect to the use of mercury in this disease, we believe that it generally proves injurious."

Dr. Dunglison says that the common remark, that if the disease proves obstinate it will yield, provided we can only 'touch the

* Vide Diseases of Bengal, by W. Twining, Esq., p. 40.

mouth' with a mercurial, does not accord with his experience. He has seen many cases in which the effect of mercury has been induced on the system, and, nevertheless, the disease has proceeded to a fatal termination. (*Practice of Medicine*, vol. I. p. 114.)

Dr. Batchelder, in the N. Y. Journal of Medicine for July 1851, confirms these statements by adding his own reliable testimony against the calomel practice.

But it is useless to multiply authorities upon this subject. I will add a case or two in illustration from my case-book :—

CASE I. Was called in to see M. J. on 3d of June, 1849—a feeble man, æt. 30, who had recently been severely ptyalised from taking six grains of calomel for a slight diarrhœa. Found him with high fever, nausea, thirst, urgent tormina and tenesmus, pain upon pressure along the course of the colon, and dejections of blood and mucus every few minutes. *His gums were swollen and sore, and he was discharging saliva freely, accompanied by intense mercurial fetor.* I bled him to syncope, had him freely leeched, and administered ℥ii. of Hope's Nitrous Acid Mixture every three hours. He was almost entirely well in two days.

I have no question but that calomel in this case was the immediate cause of the dysentery.

CASE II. Mrs. W. a robust lady, æt. 50, was seized with simple looseness of the bowels with slight griping, June 10th, 1851. Saw her on 11th,—fever intense, pulse rapid, soft, compressible, skin dry—thirst urgent—nausea and vomiting—pain upon pressure over abdomen—bowels moved every ten minutes, with such tenesmus as to force the patient to cry out—dejections pure blood. Venesection *ad deliquium*—Laudanum enema—Hope's mixture.

12*th.* No improvement—suffering excessive—experiences some relief as long as the enema is retained—still vomiting—ordered hot fomentations. ℞. Cupri Sulph. gr. iij.; Pulv. Opii gr. vi. M. Pil. 12—S. One pill every three hours, and continue enemata.

13*th.* As yesterday—probably a little less straining at stool—applied Emp. Canth. to abdomen; continue pills.

14*th.* Symptoms not quite so urgent—less nausea and thirst, but still suffers intensely. ℞. Calomel gr. xii. Ipecac. gr. iv. Morph. Sulph. gr. ij. M. Pil. 8—S. One pill every two hours.

*15th.* More fever, and dejections more frequent—took all the pills—stomach very irritable—pulse frequent and feeble—ordered laudanum enema, to be repeated whenever the bowels are moved.

*16th. Found my patient profusely salivated*, with no abatement of the dysenteric symptoms. From this time kept her upon large doses of acetate of lead and opium with laudanum injections, and occasionally a full dose of Dover's powder at night. She was well on the 22d.

These two cases taken indiscriminately from a number that have fallen under my notice, show conclusively that the mercurial impression is not incompatible with the existence of acute dysentery, and that calomel is very far from being the Magnus Apollo in the cure of this disease, which its zealous advocates claim for it.

*Saline Cathartics.*—I have prescribed a combination of Epsom and Rochelle Salts, ʒij. of each, after general bloodletting, with the happiest effects in many cases of acute dysentery—and am disposed to think very favorably of the practice. I think it is particularly applicable to cases occurring in robust persons where the tenesmus is very troublesome. Frequently have I seen this symptom entirely relieved after the copious watery evacuations produced by the saline.

*Acids.*—The nitrous acid mixture of Dr. Hope,* which was introduced to the profession in this country through the columns of this Journal, by Dr. C. D. Meigs, in 1838, is justly considered by those who have used it freely, a remedy of great value in dysentery. Among all the good things which Dr. Meigs has done for his brethren, he never did a better than calling their attention to this remedy. In my hands it has rarely failed to accomplish every thing that was desired. It is not a specific, but if fairly tried I think will less often bring disappointment than any other single remedy in this frequently intractable affection. Great care should be taken not to administer it in too small doses, an adult should not take less than ℥ii. at a time, and it should be continued at intervals of three or four hours until about eight doses have been given.

*℞. Acid Nitrosi ʒi. Tr. Opii gtt. xl.; Aquæ Camph. ℥viii. M.

*Opium and acetate of Lead.*—Since the publication of Dr. Batchelder's valuable article, before referred to, I have given the acetate of lead and opium a fair trial, and am prepared to speak as favorably of the combination as Dr. B. has done. My experience with it has been in those cases chiefly where Hope's mixture failed. I usually give as the first dose, one grain of opium with four of the acetate, and then half grain of the one with two grains of the other *immediately after each evacuation*, no matter if the bowels are moved every five minutes. Usually but a few doses are taken before relief is obtained.

*Nux Vomica.*—When the intensity of the acute stage has been somewhat lessened by proper antiphlogistic treatment, and the disease seems disposed to assume the chronic form, a combination of nux vomica with ipecac and opium will be found of great service. I usually give to an adult the following: ℞. Ext. Nucis Vomicæ gr. viii. Pulv. Ipecac gr. vi. Pulv. Opii gr. x. M. Pil. 12. S. One pill three times a day. Some cases which resisted every other mode of treatment seemed to be speedily relieved by this.

*Local Remedies.*—Blisters in dysentery have generally disappointed me—applied too early, they add materially to the excitement, not only because they are decidedly stimulant, but because they annoy the patient when called so frequently to stool—apart from this, they prevent the proper examination of the abdomen by pressure. They may be of service in the latter stage of the disease, but I doubt, after all, if the benefit obtained is a compensation for the inconvenience they occasion.

*Injections of Laudanum* are valuable auxiliaries, especially when the inflammation is seated low down in the bowel and the straining at stool is urgent. Not less than two teaspoonfuls of laudanum should be given in *as small a quantity as possible* of thin starch, and repeated if it is not retained. The nitrate of silver enemeta have also been highly extolled, but they have proved valueless in my hands in the acute stage—due probably to the fact that the first impression of the caustic is irritant, which obliges the bowel to eject it before its specific effect is produced. In the chronic stage, when the mucous membrane is less irritable, lunar caustic injections will be found to answer a good purpose, especially in the case of children. This remark applies with two-fold force to those cases where ulceration of the bowel is suspected.

*Diet.*—All medication in dysentery will prove abortive if proper attention is not paid to the regimen. Among the most important points to be observed is *not to permit the patient to take cold drinks.* I have frequently seen all the symptoms aggravated after taking a small draught of ice water, or eating a few pieces of ice. Warm sage tea answers well for the ordinary drink, usually alleviates the thirst, and is not unpalatable to most persons. Warm mucilaginous drinks, such as the gum arabic, slippery elm, &c., are also of positive service--not only because they are nourishing to some extent, but also because they shield the inflamed bowel from irritating secretions.

During convalescence the blandest food should be taken, such as arrow-root, tapioca, soft boiled eggs, &c., and these in very small quantities. I am satisfied that convalescence is often retarded, and even a relapse induced, by putting into the stomach too much even of the most wholesome food. The digestive powers are especially feeble after an attack of dysentery, and one, who has not repeatedly witnessed the fact, would scarcely believe that a single teaspoonful too much, even of the most unirritating food, will sometimes re-produce the distressing symptoms of the disease. We can not be too cautious then in reference to the *quantity* as well as the *quality* of the diet.

During the months of May and June, dysentery prevailed to a considerable extent in this city and the adjoining counties—there was scarcely a sufficient number of cases, however, to make it epidemic. The character of the disease was decidedly sthenic—the only exceptions being in those persons who had but recently recovered from measles, or whose health was impaired by an attack of fever. The lancet, leeches or cups, Hope's mixture, opium and acetate of lead, saline cathartics, and Dover's powder were used with almost uniform success in the acute stage; and the nux vomica with opium and ipecac., the vegetable astringents combined with tonics, and proper regimen, were relied on in the chronic stage. Dr. A. T. Foster, an intelligent physician of Norfolk county, informed me that he had used creasote and oil of turpentine with good result in some intractable chronic cases. He mentioned one case which well illustrates the importance of a rigid attention to the quantity of nourishment to be given. He commenced with a single teaspoonful of milk three times a day, and was unable for several days to increase the quantity—if he gave two teaspoonfuls, for instance, the dysenteric symptoms would be increased, and he could detect in the stools shreds of coagulated milk undigested. The patient at last was able to drink a pint of milk every day. He doubtless had ulceration of the mucous membrane of the colon.

