# Errata

**Published:** 1852-11

**Authors:** Geo. L. Upshur

**Affiliations:** Norfolk, Va.


### ERRATA.

*To the Editors of the Medical Examiner:*—In my article on *Dysentery*, in the September number of the *Examiner*, the types make me perpetrate some very bad grammar. Page 571, 20th line from the top, for *is* read *are*. Page 572, 23d line from top, there is a sentence which reads thus: "I can call to mind at least half a dozen cases of death from this disease, in the commencement of my practice, *which, after reflection, convinces* me might possibly have been saved by copious blood-letting." The part of the sentence italicised should have no comma, and there should be a hyphen between *after* and *reflection*, thus: "I can call to mind at least half a dozen cases of death from this disease, in the commencement of my practice, *which after-reflection convinces* me might possibly have been saved by copious blood-letting."

Yours. Geo. L. Upshur.

*Norfolk, Va.*, Sept. 9th, 1852.

